# Efficacy of dementia prevention awareness campaigns in the general population: A systematic review

**DOI:** 10.1002/alz70860_103510

**Published:** 2025-12-23

**Authors:** Jose M Aravena, María Ignacia Hamilton, Fiorella Giordano, Javiera Rodriguez

**Affiliations:** ^1^ Facultad de Medicina Clínica Alemana, Universidad del Desarrollo, Santiago, Region Metropolitana, Chile; ^2^ Department of Social & Behavioral Sciences, School of Public Health, Yale University, New Haven, CT, USA; ^3^ Facultad de Medicina Clínica Alemana, Universidad del Desarrollo, Santiago, Región Metropolitana, Chile

## Abstract

**Background:**

Although about 45% of the risk of dementia could be modifiable, nearly 80% of the population still believe dementia is a normal part of aging. Dementia prevention awareness campaigns could be low‐cost feasible strategies to promote dementia risk reduction. Nonetheless, what are their real impacts and what strategies may work better have not been analyzed. Thus, this was the aim of our study.

**Method:**

A systematic review of literature published up to July 2024, with no language or publication time restrictions was conducted. Studies in any population, using any research design, and assessing the effect of a dementia prevention awareness strategy were included. The literature search was conducted through five databases (PubMed, Scopus, Google Scholar, Lilax, Scielo). Duplicate documents were removed using EndNote, and relevant studies were selected through blinded screening by two independent reviewers and using an AI (ASReview). Results were analyzed using a narrative synthesis and studies' risk of bias.

**Result:**

From 3,898 articles retrieved, 16 studies comprising a total of 34,441 participants fulfill inclusion criteria and were included. Campaigns were tested mostly in Europe (43.8%), Australia (31.3%), and United States (17.6%), and included people from all ages (<24y: 50.0%, 24‐59y: 87.5%, >60y: 81.3%). Most of the studies were quantitative (cross‐sectional:3; pre‐posttest:9; RCT:2). The most used awareness strategies were web pages (50.0%), community talks (43.8%), and printed material (37.5%). In the group of studies using cohort designs (pre‐posttest and RCTs: 11), most of the studies found an increase in population’ knowledge about risk factors or protective behaviors (n:7/8), intention to change behavior (n:2/2), and knowledge about dementia (n:1/1). However, no consistent results were observed in dementia risk‐reduction awareness (n:3/6) and no studies assessed health behavior change. Positive results are seen most often at short‐term and using instructive strategies (MOOC, talks). One study assessed people at risk of dementia, with no significative differences. Overall, studies’ risk of bias was high.

**Conclusion:**

Dementia prevention awareness campaigns could increase knowledge about dementia risk factors across the lifespan. Nevertheless, the effect on dementia risk‐reduction awareness and behavioral change is uncertain, and more high‐quality studies targeting populations at risk of dementia are needed.

